# Stepped Coastal Water Warming Revealed by Multiparametric Monitoring at NW Mediterranean Fixed Stations †

**DOI:** 10.3390/s20092658

**Published:** 2020-05-06

**Authors:** Nixon Bahamon, Jacopo Aguzzi, Miguel Ángel Ahumada-Sempoal, Raffaele Bernardello, Charlotte Reuschel, Joan Baptista Company, Francesc Peters, Ana Gordoa, Joan Navarro, Zoila Velásquez, Antonio Cruzado

**Affiliations:** 1 Instituto de Ciencias del Mar–CSIC, 08003 Barcelona, Spain; jaguzzi@icm.csic.es (J.A.); batista@icm.csic.es (J.B.C.); cesc@icm.csic.es (F.P.); joan@icm.csic.es (J.N.); 2 Centro de Estudios Avanzados de Blanes–CSIC, 17300 Blanes, Spain; gordoa@ceab.csic.es; 3 Stazione Zoologica Anton Dohrn, 80122 Naples, Italy; 4 Coastal Dynamics Laboratory, Universidad del Mar, Puerto Ángel, 70902 Oaxaca, Mexico; ahumada@angel.umar.mx; 5 Department of Earth Sciences, Barcelona Supercomputing Center, 08034 Barcelona, Spain; raffaele.bernardello@bsc.es; 6 Department of Chemistry and Biology, Hochschule Fresenius University of Applied Sciences, 65510 Idstein, Germany; charlotte.reuschel@gmx.de; 7 Oceans Catalonia International SL, 17300 Blanes, Spain; zoilavf47@gmail.com (Z.V.); acruzado@oceans.cat (A.C.)

**Keywords:** marine-observing systems, multiparametric buoy profiling, long time-series, water warming, oligotrophy

## Abstract

Since 2014, the global land and sea surface temperature has scaled 0.23 °C above the decadal average (2009–2018). Reports indicate that Mediterranean Sea temperatures have been rising at faster rates than in the global ocean. Oceanographic time series of physical and biogeochemical data collected from an onboard and a multisensor mooring array in the northwestern Mediterranean Sea (Blanes submarine canyon, Balearic Sea) during 2009–2018 revealed an abrupt temperature rising since 2014, in line with regional and global warming. Since 2014, the oligotrophic conditions of the water column have intensified, with temperature increasing 0.61 °C on the surface and 0.47 °C in the whole water column in continental shelf waters. Water transparency has increased due to a decrease in turbidity anomaly of −0.1 FTU. Since 2013, inshore chlorophyll *a* concentration remained below the average (−0.15 mg·l^−1^) and silicates showed a declining trend. The mixed layer depth showed deepening in winter and remained steady in summer. The net surface heat fluxes did not show any trend linked to the local warming, probably due to the influence of incoming offshore waters produced by the interaction between the Northern Current and the submarine canyon. Present regional and global water heating pattern is increasing the stress of highly diverse coastal ecosystems at unprecedented levels, as reported by the literature. The strengthening of the oligotrophic conditions in the study area may also apply as a cautionary warning to similar coastal ecosystems around the world following the global warming trend.

## 1. Introduction

Global ocean warming is escalating at unprecedented rates [1], making the marine environment and living resources come under increasing stress. The five warmest years on record have occurred since 2015 [2]. The World Meteorological Organization (WMO) reported a global temperature raising of 0.2 °C for the period 2015–2019, compared to the period 2011–2015 [3] (~0.02 °C y^−1^). Though high temperature anomalies in 2015 and during the first semester of 2016 were explained by a relatively strong El Niño event [4], temperature anomalies in 2017 and 2018 were mostly not attributable to El Niño. The National Aeronautics and Space Administration (NASA), the National Oceanic and Atmospheric Administration (NOAA), and the WMO all emphasize that the 5-year period 2015–2019 has been the warmest of the last 140 years [5].

In this context, the Mediterranean Sea has also been reported as becoming warmer in the last 40 years at rates of 0.0015 °C yr^−1^ [6], though intermediate waters may be increasing at rates of 0.03 °C yr^−1^—that is, one order of magnitude greater than global ocean intermediate layer warming [7]. In a coastal area of the NW Mediterranean, an increasing trend of 0.014 °C yr^−1^ has been reported from 1974 to 2006 [8]. Long-lasting positive extreme temperatures have caused mass-mortality events of invertebrates in the Mediterranean Sea [9] with direct impact on gorgonians [10], octocorals [11], and bivalves [12] over the continental shelf. Water temperature may be exceeding the upper thermal thresholds of living organisms [11], especially affecting survival, growth, and reproduction of benthic sessile species [13,14]. Mass mortalities of sponges may be directly linked to increasing time exposure to warm temperature anomalies [15] producing a shift in presence from slow-growing to more resilient species [16]. In fact, for the NW Mediterranean, the upper limit of the thermocline layer in summer is very shallow, at around 30-m depth [17]. Thus, a sea surface warming is expected to significantly impact on life cycles of relatively shallow-living pelagic and seafloor organisms. Ocean warming has been linked with the expansion of oligotrophic areas [18], reinforcing the thermocline, and making the mixed layer thicker, as reported for the Mediterranean Sea [17]. The strengthening of the thermocline may slow down the supply of nutrients from deep waters to the euphotic zone, thus decreasing the overall phytoplankton biomass production [17,19]. A longer-lasting summer thermocline may also delay the vertical mixing of the water column in autumn and winter, which in turn constrains the winter phytoplankton bloom to shorter periods [20]. An increased frequency of mass mortalities of benthic invertebrates is likely to come in the next years [19].

Within the current context of global ocean warming, marine-observing systems with highly integrated multidisciplinary monitoring of physical and biogeochemical water properties is of strategic relevance to understand the impact of climate change and anthropic impacts on biological communities at all depths of the ocean [21,22,23,24,25]. Long-term coastal monitoring stations collecting those data from the water column are fundamental to assess the effect of environmental changes on the marine ecosystem, which in turn play a significant role in controlling the climate [26]. For example, in the Ligurian Sea, the open-sea fixed station DYFAMED has been operating since 1991 [27] collecting biogeochemical data—apart from physical water properties. Similarly, atmospheric, oceanographic, and biogeochemical data were provided during a decade (from 2009 to 2018) from two fixed coastal stations by the deep-water, coastal, moored Operational Observatory of the Catalan Sea (OOCS) at the head of the Blanes submarine canyon in the Balearic (Catalan) Sea [28]. The marine-observing systems constitute networks (e.g., EuroGOOS) providing real-time information to the public, e.g., scuba divers, fishermen, or recreational sailors. The environmental authorities dispose of this information to control, for example, water quality, pollutant dispersal, and marine safety. The networks fuel marine and atmospheric models for validating model outputs, generating reanalysis products, and improving model predictions [29].

The aim of this work is to show the potential of highly integrated multiparametric measurements of atmospheric, oceanographic, and biogeochemical data for assessing temporal changes of the water column characteristics of the periods before and after the intensified water warming starting in 2014–2015. The measurements were carried out continuously in the Balearic Sea, at a high frequency and remotely over a decade (i.e., from 2009 to 2018) at a sampling station of the observatory OOCS [28] with a mooring array in a deep-sea coastal area at the Blanes canyon head (220-m depth) and a nearby inshore sampling station. With our singular sensor platform and data collection strategy, we could evidence the extent at which that local water warming is in line with the described global warming pattern. We picture a potential scenario of impacts in the Balearic oligotrophic ecosystem, which may be mirroring the patterns of change already reported in similar coastal areas worldwide.

## 2. Materials and Methods

### 2.1. In Situ Data

**Figure 1:**
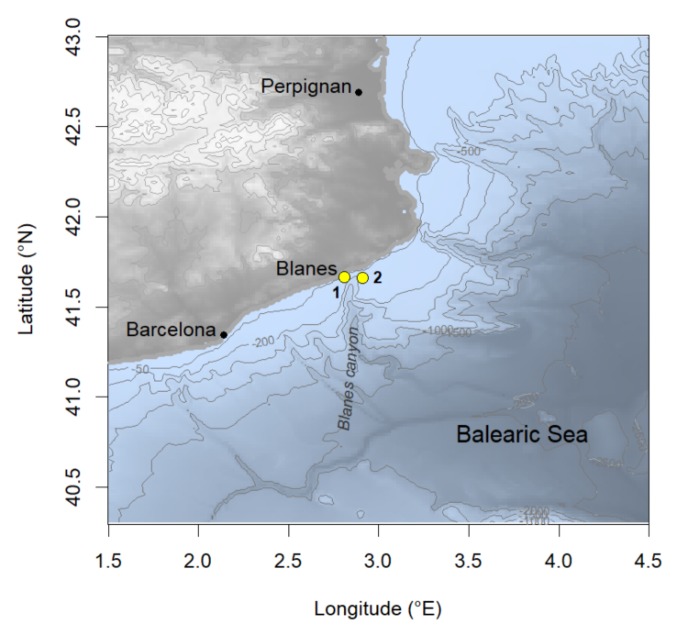
Sampling stations of the Operational Observatory of the Catalan Sea (OOCS). The inshore station (1) was located at 41.67° N, 02.79° E. The Blanes canyon station (2), equipped with an anchored oceanographic buoy, was located at 41.66° N, 02.90° E.

We processed water column time series data of physical and biogeochemical variables from two fixed monitoring stations in typically oligotrophic coastal waters of the Balearic Sea, NW Mediterranean, from March 2009 to March 2018. The stations were maintained by the OOCS [28] in the Blanes bay (hereinafter inshore station), located 0.5-nm offshore with a 20-m bottom depth; and in the Blanes canyon head (hereinafter Blanes canyon station), located 2.7-nm offshore with 230-m bottom depth (Figure 1).

The data were collected at two different time scales and methodologies. On a fortnightly basis, the two stations were visited with the research boat “Dolores” (see details in [28]). During the sampling, CTD (i.e., Conductivity, Temperature, and Depth) profiles were firstly performed from the surface down to 20 m at the inshore station and down to 230 m at the Blanes canyon station. The CTD was also equipped with fluorescence and turbidimeter. The CTD data were 1-m bin-averaged. Details of the CTD sensors are provided in [28]. Then, water samples were collected with Niskin bottles at discrete sampling depths of 1 m and 10 m at both stations, whereas at the Blanes canyon station, the sampling depths extended through the moored platform line at 25, 50, 75, 100, 140, and 185 m. The samples allowed determining concentrations of nitrate, nitrite, phosphate, and silicates along with chlorophyll *a* concentration as a proxy for phytoplankton biomass.

Water samples were processed at the Centro de Estudios Avanzados de Blanes (CEAB-CSIC) facilities. Dissolved inorganic nutrients (nitrate+nitrate (hereafter Nitrate), ortophosphate, orthosilicate acid (hereafter silicate)) concentrations were determined from 20-mL water samples. Samples were processed using an autoanalyzer Skalar, following [30]. Chlorophyll *a* concentration was determined on nonfractionated particulate matter from three-liter water samples, filtered through 4.7-cm Whatman GF/F (glass microfiber) filters and kept in the refrigerator for 24 h in 5-mL acetone (90%). After 30 min centrifugation, the absorbance was read at 750-, 664-, 647-, and 630-nm wavelength. The chlorophyll concentration was estimated following [31].

Hourly data were also collected from meteorological and oceanographic instrumentation in an oceanographic platform anchored from 15 Oct 2009 to 20 Dec 2016 at the Blanes canyon head (see details in [28]; see Figure 1). The meteorological data consisted of air temperature (°C), relative humidity (%), wind speed (m s^−1^) and direction (deg.), atmospheric pressure (bar), and photosynthetically active radiation (PAR). The temperature (°C) and salinity (psu) data were collected with three CTDs attached to the mooring line at 1-, 25-, and 50-m depth. In addition, the CTD attached at 25 m and 50 m also measured fluorescence chlorophyll (for units, see Section 2.2.), turbidity (FTU), and PAR [14]. A current-meter profiler was also attached (facedown) to the buoy to measure current’s speed (m/s) and direction (deg.) from the surface down to 80 m, with a 12-m vertical resolution (current meter data were not presented here). Owing to buoy and instrumentation maintenance, data collection was not continuous (Table 1). The buoy remains missing since a sea storm detached it from the mooring site on 20 Dec 2016.

### 2.2. Derived Variables and Auxiliary Data

**Figure 2:**
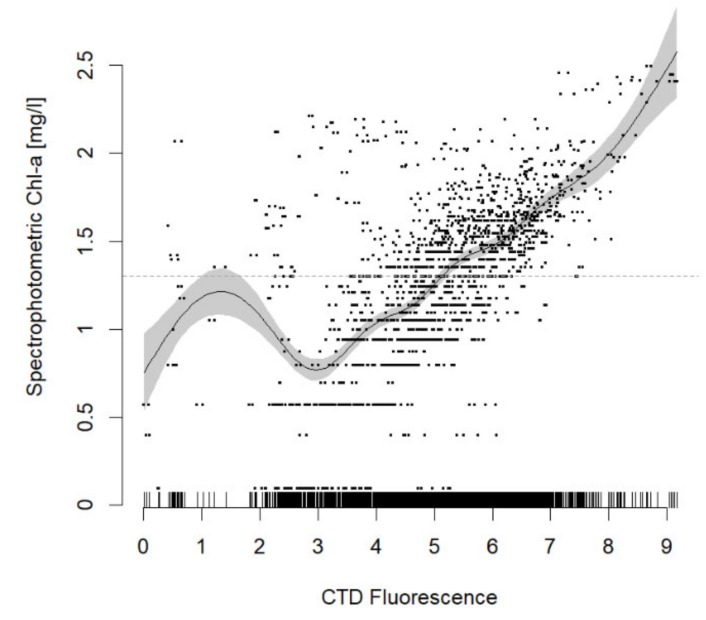
Generalized Additive Model (GAM) regression for calibrating Conductivity, Temperature, and Depth (CTD) fluorescence from spectrophotometric chlorophyll, as obtained from water samples at discrete depths in OOCS stations (*p*-value < 0.05).

CTD fluorescence was transformed to chlorophyll *a* biomass (Chl, mg l^−1^), taking as a reference the spectrophotometric chlorophylls measured in the lab from bottle samples by fitting a Generalized Additive Model (GAM) in the form of log(Chl) ~ *s*(log(Fluo)), assuming a Gaussian family distribution and a link identity. The *s* represented the smoother (polynomial) term that substitutes the slope in a linear regression (Figure 2). To avoid overfitting, a gamma correction factor of 1.5 was applied [32].

In order to assess for a synchronicity pattern between local and mesoscale Mediterranean water temperature conditions, time series from 4-km-resolution remote sensing sea surface temperature (SST) data (from the Moderate Resolution Imaging Spectroradiometer; MODIS) were processed via NASA Earth Data [33]. Yearly global temperatures were also obtained from NASA (https://climate.nasa.gov/).

Based on biweekly CTD profiles data, the mixed layer depth (MLD) was estimated following threshold methods. The depth at which the water temperature differed by 0.2 °C from the temperature at a subsurface reference depth of 10 m was assumed to be the depth of the mixed layer [34]. For comparison, the depth at which the water density differed by 0.03 kg m^−3^ from the density at the reference depth of 10 m was also assumed to be the depth of the mixed layer [35]. An approach to the duration of winter mixed events reaching depths greater than 100 m was made, assuming that two consecutive events detected two weeks apart corresponded to a single event.

For the periods in which the multiparametric buoy remained operational (see Table 1), hourly-basis data collected from meteorological and oceanographic instrumentation in the buoy, i.e., air temperature and humidity plus wind speed, atmospheric pressure, and SST (Figure S1), were used to estimate daily net surface heat flux (*Qt*), from the radiative incoming and leaving heat fluxes, following the equation

*Qt* = *Qs* – *Qb* − *Qe* – *Qh,* (1)

where, *Qs, Qb, Qe,* and *Qh* represent the short-wave radiation, the long-wave radiation, the latent heat flux, and the sensible heat flux, respectively. Each one of the terms in the equation were solved following the procedures indicated in Appendix A. The restriction of *Qt* estimates to different periods per year (i.e., incomplete years, see Table 1) prevented proving yearly estimations of *Qt*. Therefore, daily *Qt* from reanalysis products from NOAA [36,37], covering complete years (2009–2016) were used for comparison with in situ data and for estimating average yearly *Qt* values for the Blanes canyon area.

## 3. Results

### 3.1. Water Temperature and Salinity

**Figure 3:**
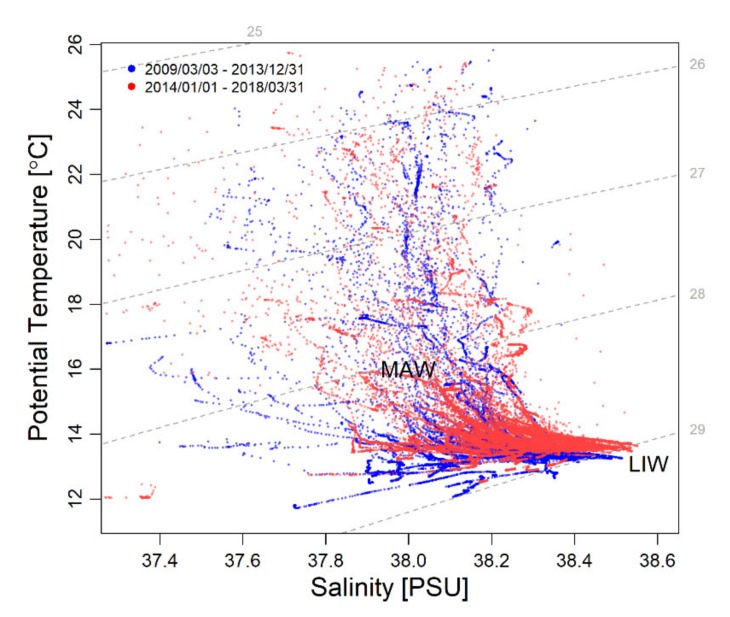
Temperature–Salinity (TS) diagram showing values recorded since March 2009 to March 2018 at the Blanes canyon head. Measurements collected before and since Jan 2014 are represented in blue and red, respectively Grey dashed lines are the sigma-*t* values, i.e., water density in kg m^-3^ – 1000 (see Figure S2).

The water column over the edge of the continental shelf in the Blanes canyon station (from surface to 230 m depth), showed temperature ranges from 11.73 °C in winter to 25.78 °C in summer, whereas salinity ranged from 37.3 to 38.5 psu, the values were generally found to be lower in summer and higher in winter (Figure S2). The seasonal changes were more noticeable in the upper 150-m depth of the water column, composed by Modified Atlantic Water (MAW) (Figure 3). The lower water layer below 150-m depth and reaching the shelf bottom was more stable over the whole year and showed characteristics closer to the Levantine Intermediate Water (LIW) (Figure 3) formed in the eastern Mediterranean Sea basin.

**Figure 4:**
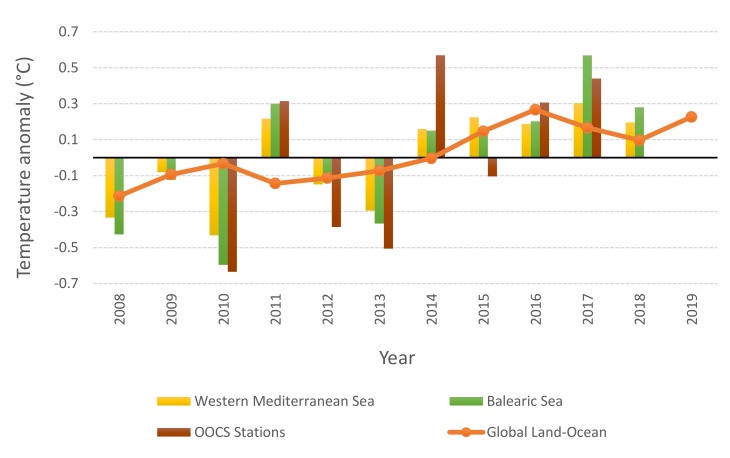
Temperature anomalies relative to 2009–2018 base period at different spatial scales, from local data at the OOCS stations to global data.

The analysis of yearly SST anomalies (i.e., from 2- to 20-m depth including complete sampling years from 2010 to 2017) at the two OOCS monitoring stations revealed a sustained climb starting in 2014 above the average (Figure 4). Both stations showed an average SST for the period 2014–2017 of 0.61 °C higher than the average for the period 2010–2013 (Figure 4). Such a sudden SST increase was also detected along the water column down to 200-m depth. The average temperature over the water column was 0.47 °C higher during 2014–2017 than during 2010–2013.

The analysis of yearly SST anomalies over the western Mediterranean Sea for 2008 to 2018 revealed a pattern similar to that found at a local OOCS station’s measurement scale (Figure 4). The Balearic SST showed a 0.49 °C increase in 2014–2018 compared to 2008–2013, whereas the western Mediterranean basin showed a difference of 0.39 °C between the two periods. The reported global land–ocean temperatures from 2008 to 2018 reveal an increasing SST of 0.25 °C for 2013–2018 compared to 2008–2013, this even increases to 0.26 °C if 2019 is included (Figure 4)**.**

The yearly water column salinity was rather constant during the study period (Figure S2), remaining around 38.14 ± 0.07 psu (mean ± SD) for the period 2010–2013 and 38.17 ± 0.08 psu during 2014–2017. The years 2012, 2016, and 2017 showed the highest salinity values (between 38.23 and 38.25 psu), whereas 2011 and 2014 showed the lowest yearly salinity values (38.07 and 38.08 psu, respectively).

### 3.2. Mixed Layer Depth (MLD)

**Figure 5:**
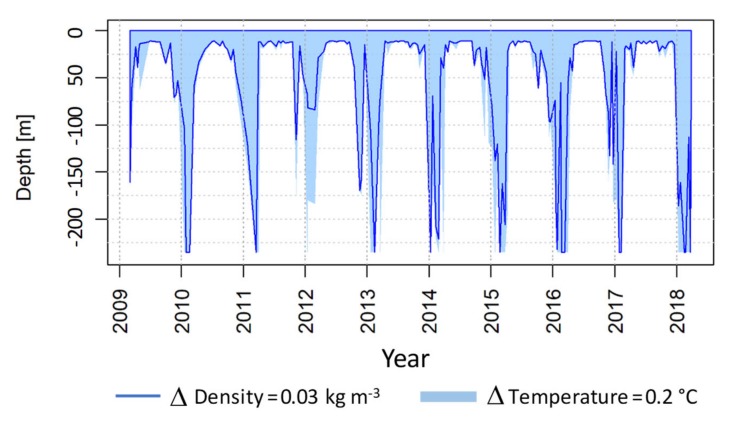
Time series of mixed layer depth (MLD) estimates based on water density and temperature thresholds.

The MLD generally reached the bottom in winter, independently of the threshold criteria (i.e., regardless of being based on water temperature or density), though it generally reached deeper depths when using the temperature criteria (Figure 5).

**Figure 6:**
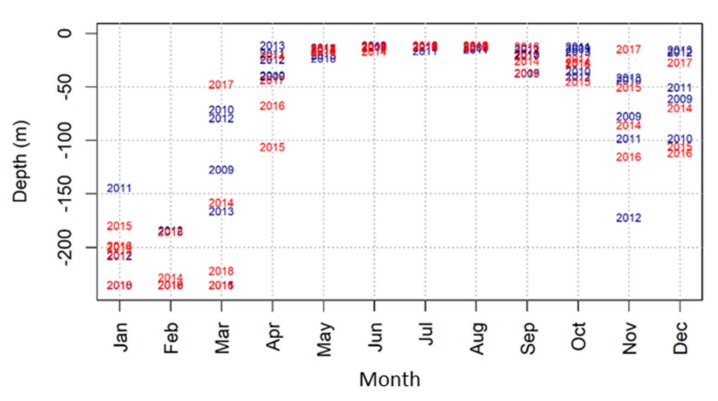
Monthly MLD based on the temperature threshold. The depth is indicated as the year of the occurrence, and colors indicate the period, 2009–2013 (blue) and 2014–2018 (red).

The summer MLD remained steady for the monitoring period. Based on the temperature threshold, the depth of the mixed layer remained relatively shallow above 25-m depth from May to August (Figure 6). The highest interannual variability of the MLD was observed in March (fluctuating from 50- to 200-m depth) and in November and December (fluctuating between 20- and 170-m depth). For the particularly warm years of 2015 and 2016, the MLD in April went deeper—between 70- and 105-m depth—than any other year in the time series, showing depths between 15- and 48-m depth.

**Figure 7:**
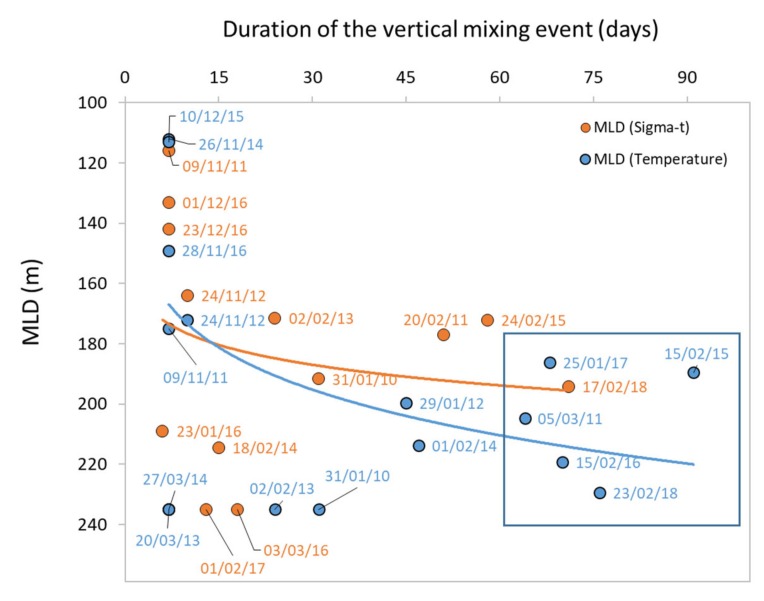
Relationship between the winter mixed layer depth (MLD) placing itself below 100 m and its duration (in days). The relationship is stronger (i.e., higher *r^2^*) when MLD is estimated from temperature threshold (*MLD* = 135.28 *Duration* ^0.1079^, *r^2^* = 0.22) than when estimated from sigma-t threshold (*MLD* = 135.28 *Duration* ^0.1079^, *r^2^* = 0.04). The blue square highlights the vertical mixing events lasting for more than two months and reaching depths greater than 180 m.

The temperature threshold criteria allowed determining a trend for the winter MLD to get deeper and lasting for longer periods with time (Figure 7). Based on the temperature threshold, mixed layer events were observed deeper than 180 m and lasting between 60 days and 90 days both during 2011 and between 2015 and 2018.

### 3.3. Biogeochemical Water Properties

**Figure 8:**
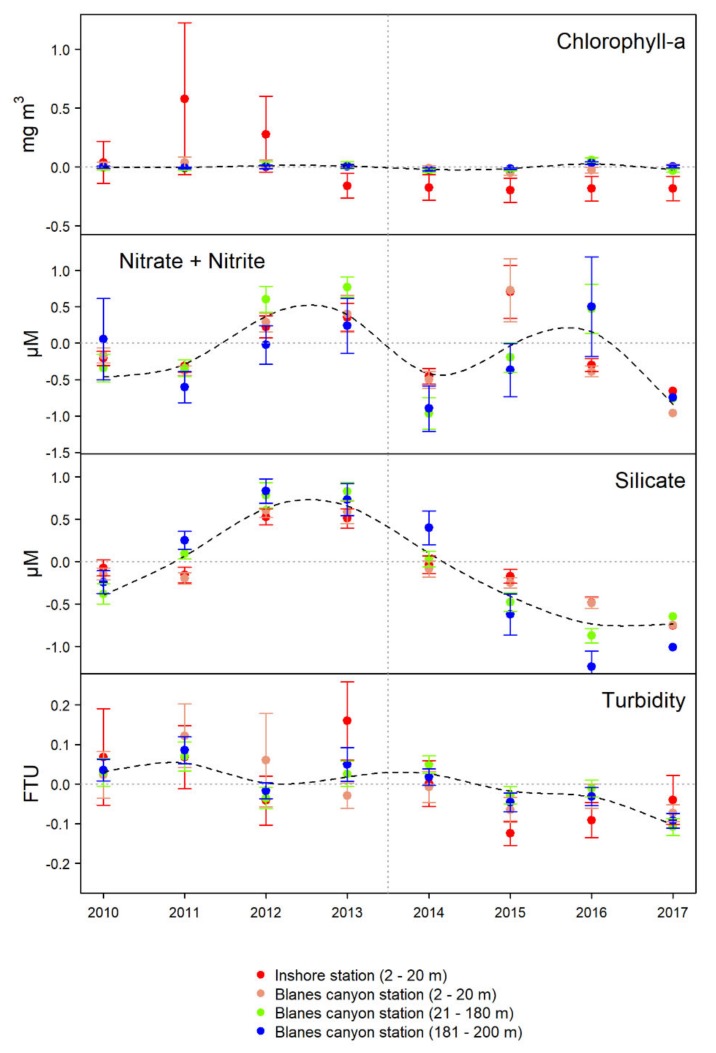
Anomalies (with error bars) of biogeochemical variables measured at different depth layers in the inshore station and the Blanes canyon station. The dotted black line represents the overall trend adjusted for the data averaged along the whole water column in the canyon station (2–200 m depth).

The yearly average chlorophyll *a* concentration (i.e., proxy of phytoplankton biomass) generally remained steady all over the water column during the study period (complete years from 2010 to 2017), particularly in the Blanes canyon head station (Figure 8, Figure S3). The inshore station showed surface chlorophylls anomalies far from the average in 2010 (anomaly of 0.6 mg l^−1^) and 2011 (anomaly of 0.3 mg l^−1^), though it remained below the average 0.15 mg l^−1^ from 2013 to 2017.

The yearly average concentration of nitrate (Figure 8, Figure S3) showed oscillations all over the water column, with minimums (values below the average) in 2011, 2014, and 2017, and maximums in 2009, 2010, and 2016. The years 2015 and 2016 showed the highest variation of nitrate concentration with depth, showing values above the average in the upper-20-m depth in 2015 and between 21–200 m depth in 2016. Silicates concentration showed a gradual increase since 2010 to 2013, and then decreased to reach the minimum values in 2016 and 2017 (Figure 8, Figure S3). The turbidity anomaly in the whole water column (i.e., 2–200 m depth) showed a trend to decrease from about 0.03 FTU in 2010 to −0.07 FTU in 2017 (~0.1 FTU), with the inshore station presenting the highest variability in 2013 (Figure 8, Figure S3).

**Figure 9:**
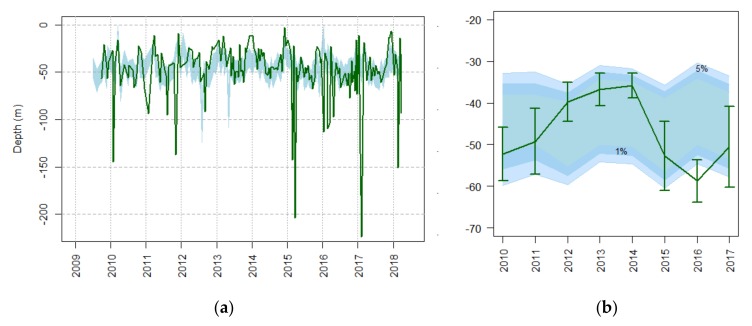
Time series of chlorophyll-*a* maximum depths (green line) compared to the lower limits of the euphotic zone (blue-shadowed area) at depths of 5% and 1% of the surface PAR depth. Fortnightly data (**a**) and yearly data for complete years (**b**) (± standard error) are provided.

The depth of the chlorophyll concentration maximum was widely variable, between 5 and 220 m depth, depending on season (Figure 9a). That depth range of the euphotic zone, i.e., the well-illuminated upper water layer where phytoplankton photosynthesis takes place, corresponds to radiation attenuation (between 1% and 5%) distance after quanta enter the sea surface (Figure 9). The deeper maximums were found from January to March (Figure 9a, Figure S2). Particular deepening of the chlorophyll-*a* maximum was observed in the winters from 2015 to 2018, making the yearly average to be located at the lower limit of the euphotic zone of 1% (Figure 9b).

### 3.4. Air-Sea Heat Fluxes

**Figure 10:**
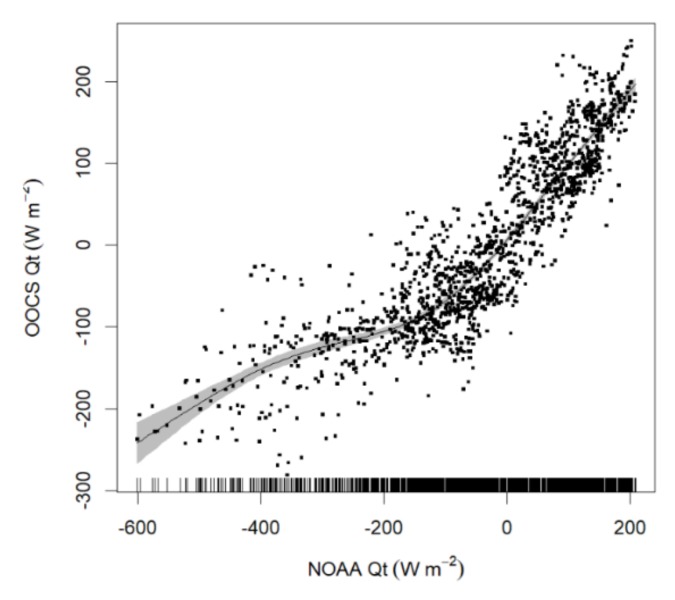
Regression using GAM, adjusted to compare National Oceanic and Atmospheric Administration (NOAA) Qt estimates with in-situ OOCS Qt estimates (*p*-value < 0.05).

The NOAA and the in situ (OOCS) net sea heat flux (Qt) values were compared by fitting a regression using GAM in the form of OOCS Qt ~ *s*(NOAA Qt), with the *s* representing the smoother function (Figure 10). The NOAA values were generally lower than the in-situ estimates (i.e., from the buoy), although they are highly correlated (*r* = 0.86, Spearman correlation). The magnitudes of the two datasets mainly diverged at negative values lower than 100 W m^-2^ (Figure 10).

**Figure 11:**
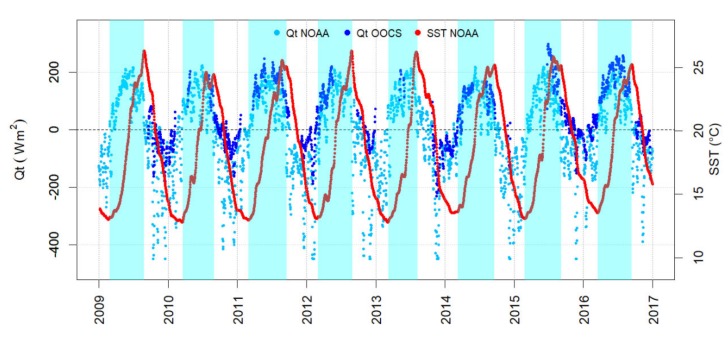
Time series of daily net air–sea heat fluxes (*Qt*) provided by NOAA (light blue solid circles) compared to the estimates from the oceanographic buoy (blue points) at the Blanes canyon station and sea surface temperature (SST) provided by NOAA (red points). Shadowed areas represent the period of the year with positive *Qt* values.

The average yearly *Qt* value from NOAA estimates between 2009 and 2016 (i.e., the period in which the buoy remained operational) was −10.2 W m^−2^, ranging from −25.9 to + 1.4 W m^−2^ (Figure 11). The dates for the starting of positive *Qt* values (i.e., *Qt* > 0) ranged from 25 Feb to 17 Mar and lasted for an average period of 181 ± 16 days (± SD), 29 Aug–18 Sep (Figure 11). For the period before 2014, the number of days with positive *Qt* was higher (177 ± 18 SD) than for the period since 2014 (188 ± 7 SD), though no significant differences between the two periods was found (*p*-value > 0.05, Mann–Whitney U-test).

## 4. Discussion

Here, we presented observations from a long-lasting multiparametric monitoring at two Balearic Sea sampling stations. Results show that average coastal SST during 2014–2017 have drastically scaled 0.61 °C above the average for the period 2010–2013. Despite being well within the natural interannual variability, this represents about 68% of the global temperature increase in a century (i.e., 0.9 °C during 1900–2013 [2]). That climbing pattern was clearly showed during the period 2014–2017 in line with the stepped warming pattern of the earth surface reported by [4] of 0.24 °C. The authors relate this global warming pattern to an unusual release of heat from the subsurface of the tropical Pacific Ocean to the atmosphere, which has been accumulating since the 1990s due to greenhouse gases. Here, we highlighted how that global temperature increase may keep a relatively fast pace, as the average temperature for 2013–2018 was 0.25 °C greater than the previous period 2008–2013, and after adding the average temperature for 2019, the average anomaly for 2013–2019 even raised 0.1 °C (i.e., 0.26 °C).

The temperature anomaly in the sampled stations follows the general Mediterranean trend (i.e., +0.39 °C for the western Mediterranean SST and +0.49 °C for the Balearic SST). More importantly, that warming may not be restricted to the sea surface but extending to the water column over the continental shelf (i.e., 0.47 °C, present work). There is a consensus of the warming and salting trends of the Mediterranean Sea [6,7,27]. The trend may show abrupt changes like those reported here since 2014, and may also have oscillatory patterns of a 3- to 6-year period, as reported for a nearby shallower (80-m depth) coastal observation station [8]. In the western Mediterranean, another abrupt change took place in 2005, when a combination of anomalous atmospheric forcing and a preconditioning caused by saltier intermediate waters triggered the so-called Western Mediterranean Transition (WMT), characterized by massive production of warmer and saltier deep-water masses [7].

The deepening and lasting of the winter MLD at the Blanes canyon station seems to be counterintuitive to the overall water warming, particularly in winter, when the MLD takes place. The deepening of the mixed layer is a trend observed in the Mediterranean Sea, as reported in a study reconstructing the MLD since 1945 to 2011 [17], attributed to undetermined effects of atmospheric warming, winds, and air–sea interaction. Although strong cold winds blowing over the sea surface are often responsible for a deep convection in winter (i.e., deepening of the mixed layer) [38], relatively low stratification conditions of the earlier autumn may also contribute to the deepening of the mixed layer [39]. The vertical winter convection, produced due to increasing sea surface density, can result from the prewinter conditions when salinity increases in the water column and the temperature is the result of the combination of the initial heat content of the water column and of the surface heat fluxes. Though the relatively low increasing of salt content (0.03 psu) during 2014–2017 may have contributed to the deepening of the mixed layer, the present analysis cannot further assess its relative role among other possible codivers.

Persistence of longer and warmer summers have been expected to expand oligotrophic areas of the ocean [18,40,41] with unanticipated consequences on pelagic and benthic organisms’ survival [13,14]. The analysis of water turbidity suggested a lowering with time and, therefore, an increasing water transparency, which is a signal of stressing oligotrophic conditions [18]. Climatic global predictions [4] already suggested the anomalously warming conditions recorded between 2014 and 2018 and also suggested that the anomaly may last to 2022, which could strengthen the oligotrophic conditions of coastal waters with negative influences on the biology and ecology of pelagic and seabed species. The records of massive mortalities of invertebrates are indicative of the expected consequences of pronounced oligotrophic conditions. The warmer period from 2014 to 2017 may be reinforcing the summer thermocline without increasing the MLD. This may produce a higher impact on the species living in shallower areas, as the relatively higher temperatures are restricted to the upper water layer. Nevertheless, the vertical mixing after the thermocline break at the end of summer may bring warmer water toward the bottom, thus lengthening the summer effect on local communities. This fact has been pointed out as being directly responsible for mass mortality of sponges, corals [6,15,16,17], and other invertebrates such as gorgonians [10] that are particularly sensitive to water warming.

In the Blanes canyon station, the stepped warmer conditions were not accompanied by specific yearly trends in the nutrient and chlorophyll *a* (as a proxy for phytoplankton biomass) concentrations, thus suggesting that the sea surface fertilization from deeper water layers was not compromised. Nevertheless, some constraint for the upward transport of nutrients because of the strengthening of the summer thermocline may be compensated by the winter and spring deepening of the MLD bringing nutrients up to the surface [42]. In the inshore area (inshore station), the oligotrophic pattern is more clear during 2013–2017, as water temperature was higher, the chlorophyll *a* concentration was below the average, and the nitrate concentration remained below the average (since 2014, exception made for 2015) along with a persistent declining of the silicate concentration since 2013. This pattern is in line with what is expected from water warming that may slow down the nutrients’ supply toward the surface and lessen the phytoplankton biomass [17,19,40].

The present work shows that the lower boundary of the euphotic zone (i.e., depth at 1% to 5% the surface radiation) remains within the depth range reported in the literature for the Catalan Sea, around 50–70 m [43]. Nevertheless, a relatively high seasonal variability was found, bringing the lower boundary of the euphotic zone to a wider range between 30 and 120 m depth. The upward displacement of the chlorophyll maximum from the 1% to the 5% depth of surface radiation during 2012–2013 can be explained by the relatively high availability of nutrients below the thermocline (i.e., nitrate+nitrite and silicates). The position of the chlorophyll maximum in 2014 around the 5% depth of surface radiation seems unrelated to the availability of the nutrients measured here, but to other nutrients, e.g., phosphates from aerosol depositions, that may explain up to 10% of the chlorophyll variability in the Mediterranean Sea [44]. The jump of water temperature observed since 2014 was found not to be related to the surface heat fluxes patterns, i.e., the yearly *Qt* showed magnitudes within the ranges of the interannual variability with no significant trend. After analyzing *Qt* duration, we did not found evidence of lengthening of the summer conditions, because the relatively high interannual variability prevented us to see any trend. Considering that, the main driver of surface heat fluxes is the SST [45], this mismatch between local warmer water with relatively steady *Qt* may be explained by advected offshore heat toward the moored platform. The eastern boundary of the Blanes canyon is believed to be able to deviate the Northern Current toward the continental shelf, thus providing the coastal region with offshore water characteristics [46]. According to the Mediterranean Sea net surface heat flux map [47], the mooring site at the Blanes canyon at 41.66°N, is located at the point at which the annual mean *Qt* shifts from positive (i.e., the surface water gains heat from the atmosphere) southward to negative (i.e., the surface water loss heat toward the atmosphere) northward. The yearly mean *Qt* provided here (−10.2 W m^−2^) is below the expected annual-mean *Qt* for the whole Mediterranean of − 5.6  ±  1.6 W m^−2^ [47]. Unfortunately, the sampling strategy at the monitoring stations may not be fully suited to capture the water properties’ variability produced by mesoscale processes (i.e., the Northern Current affecting the heat transport over the Blanes canyon area, such as cyclone and anticyclone eddies) that provoke upwelling and downwelling events, with about 4 to 20 days duration [46].

The intensification of the marine oligotrophic conditions [8] and the direct impact on marine life (e.g., [16,17]) is just one of the consequences of increasing rates of seawater warming. Ocean warming is also contributing to an increase in the strength and frequency of hurricanes, tropical storms, and floods [19]. Another consequence is the sea level rising at unexpected rates due to the thermal expansion and to the melting ice [20]. Weakening of the global circulation may be also be taking place, particularly in winter and spring [21], with unclear consequences for regional and global climate. Notwithstanding the importance of monitoring the physical and biochemical biological characteristics of the water column over the continental shelf, they remain undersampled because of the lack of long-term observing systems. Marine weather forecasting may benefit from increasing marine-observing systems. Long-term monitoring in coastal and open sea areas with diverse in situ and remote arrays of sensors are fundamental to better understanding at which extent the oceans are being altered by global warming and how those changes distress marine life, natural resources, and human life.

## 5. Conclusions

Marine observatories with coordinated multiparametric measurements of physical and biogeochemical properties of the air–sea interface and water column are fundamental to assess the diverse effects of water warming on the marine environment. The analysis of physical and biogeochemical conditions of the last decade of the water column over the continental shelf around the Blanes canyon mouth revealed a signal of stressing oligotrophic conditions since 2014, particularly supported by stepped water heating and the increasing water transparency. The deepening and lengthening of winter mixed layer events that seem counterintuitive with water warming require further investigation, along with their potential connection to the local currents’ patterns influencing the air–sea heat fluxes.

As the stepped warming in 2014 influenced not only the sea surface but also extended all over the water column above the continental shelf, it is expected that the biological communities inhabiting the deeper water layers are subject to stressing conditions similar to those produced on the organisms inhabiting shallower water layers and reported in the literature. Correspondingly, and despite the limited length of the time-series presented here, as the warming trend in the local area near the Blanes canyon follows regional and global warming patterns, the present findings may apply as a cautionary warning to other temperate oligotrophic areas subject to similar environmental stressors.

## Figures and Tables

**Table 1:** Periods of data collection by the oceanographic buoy at the Blanes canyon station. Number of days with data collection per deployment and without data between deployments are indicated.

Deployment Number	Initial Date	Final Date	Days per Deployment	Days Between Deployments
1	15/09/2009	08/02/2010	146	-
2	15/04/2010	28/04/2010	13	66
3	03/08/2010	19/01/2011	169	97
4	25/03/2011	03/06/2011	70	65
5	28/06/2011	04/10/2011	98	25
6	25/11/2011	25/05/2012	182	52
7	22/09/2012	29/12/2012	98	120
8	07/05/2013	31/05/2013	24	129
9	25/10/2013	28/05/2014	215	147
10	05/08/2014	22/09/2014	48	69
11	27/11/2014	10/12/2014	13	66
12	23/06/2015	07/09/2016	442	195
13	03/10/2016	20/12/2016	78	26

## Supplementary Materials

The following are available online at https://www.mdpi.com/1424-8220/20/9/2658/s1, Figure S1: Time series of meteorological and oceanographic information from the buoy instrumentation, Figure S2: Time series of physical properties of the water column from CTD profiles, Figure S3: Time series of the biogeochemical properties of the water column from Niskin bottle samples.

## Appendix A

**Air–Sea Heat Fluxes**

The short-wave radiation (*Qs*), considered the most influencing variable for calculating sea surface heat budget [47], was estimated following [48,49]:

*Qs* = *Q*0 · (1 − 0.62 · *C* + 0.0019 · *α*) (1 − *A*), (A1)

where *C* is the cloud cover (oktas), *A* is the albedo (0.06), and *Q0* is the clear-sky mean daily insolation (W m^-2^), calculated following [50]:

*Q*0 = *A*0 + *A*1 ∙ *cos* (*φ*) + *B*1 ∙ *sin* (*φ*) + *A*2 ∙ *cos* (2 ∙ *φ*) + *B*2 ∙ *sin* (2 ∙ *φ*), (A2)

where *A0, A1, B1, A2,* and *B2* were estimated following the equations:

*A*0 = 707.25 − 4.07 ∙ *L* − 0.038 ∙ *L*^2^, (A3)

*A*1 = 107.50 − 12.09 + 0.088 ∙ *L*^2^, (A4)

*B*1 = −9.90 + 5.08 ∙ *L* − 0.035 ∙ *L*^2^, (A5)

*A*2 = -2.22 − 0.96 ∙ *L* + 0.022 ∙ *L*^2^, (A6)

*B*2 = −80.08 + 5.02 ∙ *L* − 0.070 ∙ *L*^2^, (A7)

where *L* is the latitude of the station—which in the present case for the OOCS station is 41.66°N. Consequently, the coefficients for the equation were calculated pursuant to the equations for the range of latitudes from 40°N to 60°N.

The coefficient *φ* was calculated as a function of the Julian day (*t*) as follows:

*φ* = (2*π* / 365) ∙ (*t* − 21). (A8)

The noon solar altitude α in Equation (A1) is dependent on the declination (*δ*) of the sun (Equation (A9)) as well as on the latitude (*L*):

*α* = 90° − *L* + *δ*, (A9)

*δ* = 23.87 ∙ *sin* [2 ∙ *π* ∙ (*t* − 82)/365]. (A10)

The long-wave radiation (*Qb*) depends on SST and the water vapor content (humidity) above the sea surface. *Qb* was calculated according to Equation (A11) by [48,51]:

*Qb* = *σ*_S_ ∙ ε ∙ (*SST* + 273.15)^4^ ∙ (0.254 − 0.00495 ∙ *e*_*a*_) ∙ (1 − 0.7 ∙ *C*). (A11)

The value of the Stefan–Boltzmann constant *σ_s_* is given in SI units by *σ*_*s*_ = 5.67*x*10^−8^ *Wm*^−2^*K*^−4^. The parameter *ε* = 0.97 expresses the emissivity of the SST. The water vapor pressure of the air (*e_a_*, mbar), at sea level is calculated as

*ea* = (*RH* / 100) ∙ *ew*, (A12)

*ew* = 0.98 ∙ [1 + 1·10^−6^ ∙ *P* ∙ (4.5 + 0.0006 ∙ *SST*^2^)] ∙ 10^*γ*^, (A13)

*γ* = (0.7859 + 0.03477 ∙ *SST*)/(1 + 0.00412 ∙ *SST*), (A14)

where *RH* is the relative humidity, *e_w_* is the saturation vapor pressure, and *P* is the atmospheric pressure (mbar).

The latent heat flux (*Qe*) is estimated using bulk formula [48,50]:

*Q*e = *E* ∙ *L*_*V*_ = *ρa* ∙ *Ce* ∙ *W* ∙ (*qs* − *qa*) ∙ *L*_*V*_, (A15)

where *E* is the evaporation ratio, defined as the mass of water evaporated per unit area per unit time; and *L_V_* (J kg^-1^) is the latent heat of evaporation of water, which is calculated from SST:

*L*_*V*_ = 2.5008 ∙ 10^6^ − 2.3 ∙ 10^3^ ∙ *SST*. (A16)

The density of air at the sea surface level was assumed to be *ρa* = 1.25 *kg* *m*^-3^. *Ce* = 1.5 ∙ 10^−3^ is a transfer coefficient for latent heat (water vapor). The specific humidity at saturation in surface water denoted by *qs* (Equation (A17)), and *qa* is the specific air humidity (Equation (A18)). W (in m s^-1^) is the wind speed and SST is the sea surface temperature (in °C) provided by the OOCS.

*qs* = (0.62197 ∙ *e*_*w*_)/(*P* − 0.378 ∙ *e*_*w*_), (A17)

*qa* = (0.62197 ∙ *e*_*a*_)/(*P* − 0.378 ∙ *e*_*a*_). (A18)

Positive latent flux indicates heat transferred from the ocean to the atmosphere.

The sensible heat flux (*Qh*) depends on the temperature difference between the ocean and the atmosphere as well as on wind speed. *Qh* is calculated using the following bulk aerodynamic formulas (Equations (A19)–(A21)):

*Qh* = *ρa* ∙ *Cp* ∙ (0.0026 + 0.86 ∙ *W* ∙ ∆*T* ∙ 10^−3^); *W* ∙ ∆*T* < 0, (A19)

*Qh* = *ρa* ∙ *Cp* ∙ (0.002 + 0.97 ∙ *W* ∙ ∆*T* ∙ 10^−3^); 0 ≤ *W* ∙ ∆*T* ≤ 25, (A20)

*Qh* = *ρa* ∙ *Cp* ∙ (1.46 ∙ *W* ∙ ∆*T* ∙ 10^−3^); *W* ∙ ∆*T* > 25. (A21)

The temperature difference (Δ*T*, Equation (A22)) between the ocean and the atmosphere includes the temperature of the air above the sea surface level *TA* (in °C) and SST. *Cp* (*J* *kg*^−1^ °*C*^−1^) is the specific heat capacity of air at constant pressure (Equation (A23)) [48,50,52,53].

∆*T* = (*SST* − *TA*), (A22)

*Cp* = 1.0046 ∙ (1 + 0.8375 ∙ *qa*). (A23)

Positive sensible flux indicates heat transferred from the ocean to the atmosphere.
