# Supplementary material for: Stepped Coastal Water Warming Revealed by Multiparametric Monitoring at NW Mediterranean Fixed Stations
Source: Sensors (Basel). 2020 May 6;20(9):2658. doi: 10.3390/s20092658 (PMC7248696; doi:10.3390/s20092658)
Supplement: Supplementary file 1 [file sensors-20-02658-s001.pdf]

sensors

Supplementary material

Stepped coastal water warming revealed by multiparametric monitoring at NW Mediterranean fixed stations

Nixon Bahamon [1,2,*], Jacopo Aguzzi [1,3], Miguel Ángel Ahumada-Sempoal [4], Raffaele Bernardello [5], Charlotte Reuschel [6], Joan Baptista Company [1], Francesc Peters[1], Ana Gordoa [2], Joan Navarro [1], Zoila Velásquez [7], Antonio Cruzado [7]

[1] Instituto de Ciencias del Mar – CSIC, Barcelona (Spain); n.bahamon@csic.es (N.B.); jaguzzi@icm.csic.es (J.A.); batista@icm.csic.es (J.B.C.); cesc@icm.csic.es (F.P); joan@icm.csic.es (J.N.)
[2] Centro de Estudios Avanzados de Blanes – CSIC, Blanes (Spain); gordoa@ceab.csic.es (A.G.)
[3] Stazione Zoologica Anton Dohrn, Naples (Italy)
[4] Universidad del Mar (Mexico), Puerto Ángel (Mexico); ahumada@angel.umar.mx (MA.A-S)
[5] Barcelona Supercomputing Center, Barcelona (Spain); raffaele.bernardello@bsc.es (R.B)
[6] Fresenius University of Applied Sciences, Idstein (Germany); charlotte.reuschel@gmx.de (CH.R)
[7] Oceans.cat, Blanes (Spain); acruzado@oceans.cat (A.C; Z.V)
* Correspondence: n.bahamon@csic.es; Tel.: +34932309500.

Received: date; Accepted: date; Published: date

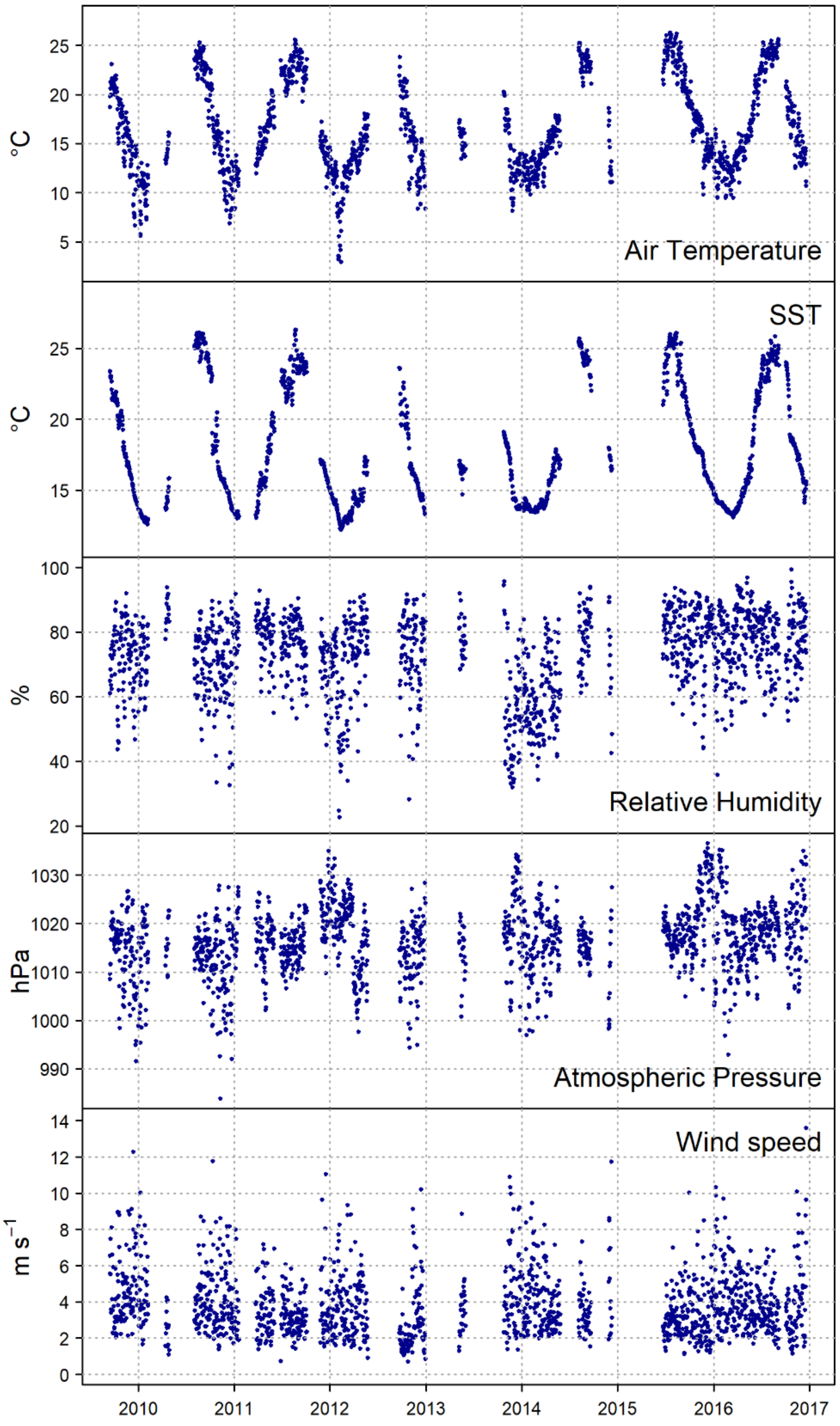

Figure S1. Time series of meteorological and oceanographic information from the buoy instrumentation.

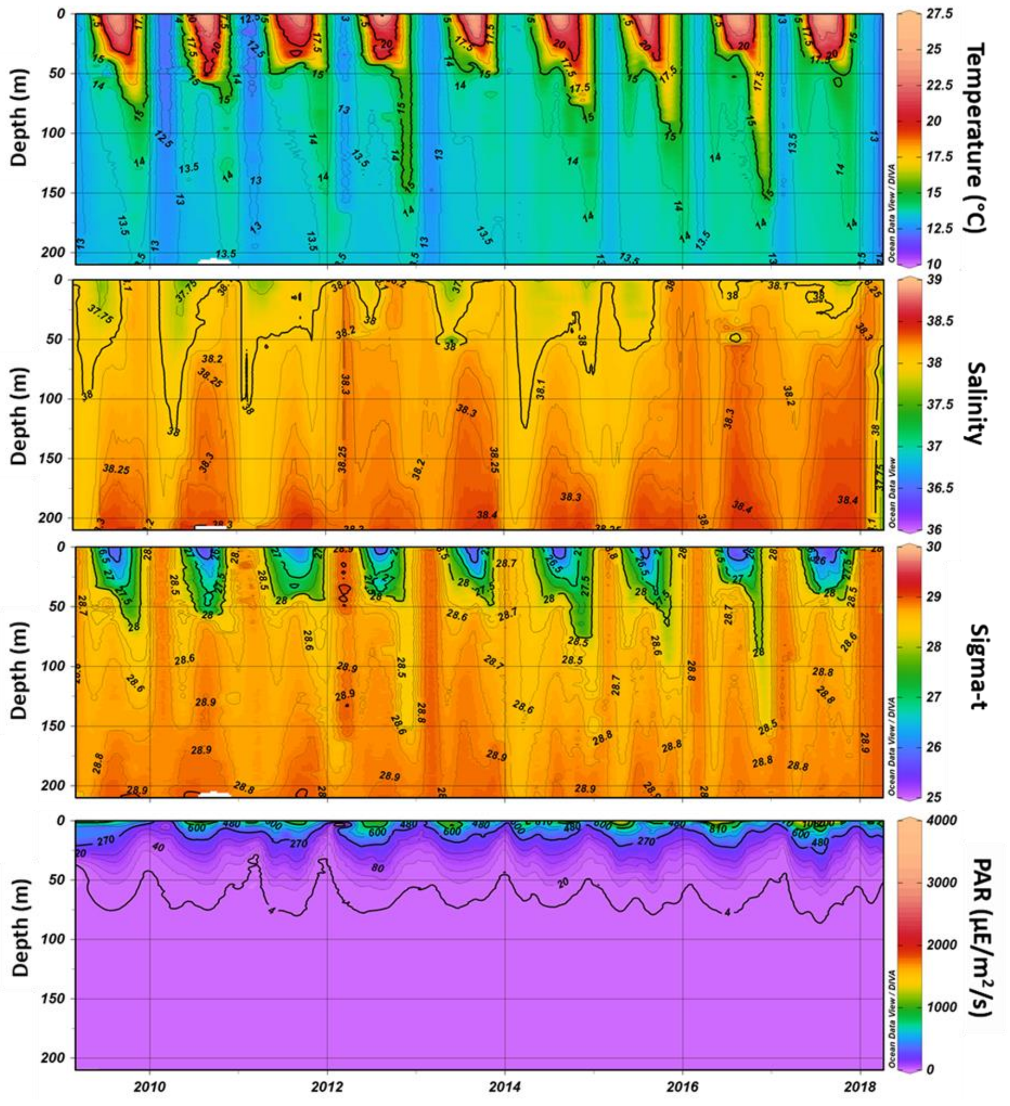

Figure S2. Time series of physical properties of the water column from CTD profiles.

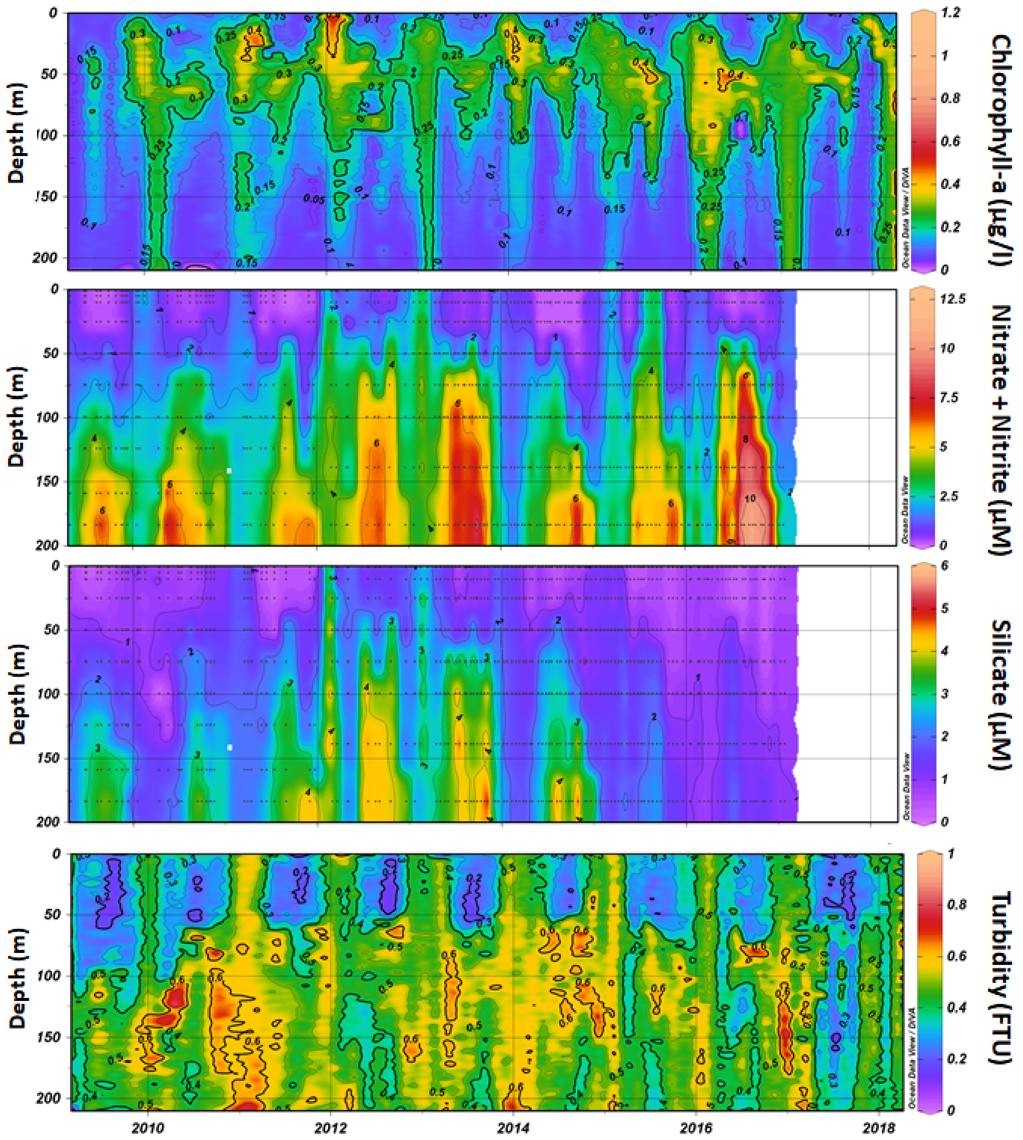

Figure S3. Time series of the biogeochemical properties of the water column from Nisking bottle samples.
